# Food—Consistency/Texture—Specific Pharyngeal Dystonia: A Novel Form of Focal Task‐Specific Dystonia

**DOI:** 10.1002/mdc3.70090

**Published:** 2025-05-06

**Authors:** Domenico A. Restivo, Daniele Bruschetta, Angelo Alito, Enrico Alfonsi, Rosario Marchese‐Ragona, Angelo Quartarone

**Affiliations:** ^1^ Department of Clinical and Experimental Medicine University of Messina Messina Italy; ^2^ Department of Biomedical, Dental Sciences and Morphological and Functional Images University of Messina Messina Italy; ^3^ Neurophysiopathology Department C. Mondino National Neurological Institute Pavia Italy; ^4^ Sleep Apnea and Dysphagia Unit, Department of Neurosciences Azienda Ospedale Università di Padova Padua Italy; ^5^ IRCCS Centro Neurolesi Bonino Pulejo Messina Italy

**Keywords:** pharyngeal dystonia, dysphagia, task‐specific dystonia, swallowing, botulinum toxin

## Introduction

Focal task‐specific dystonia (FTSD) manifests as a loss of voluntary motor control during prolonged practice of a specific motor skill that may be triggered by impairment in both sensory processing and sensorimotor integration.^1^, ^2^ The association of oropharyngeal dysphagia with dystonia is very rare, unlike that with Parkinson's disease.^3^, ^4^, ^5^, ^6^

We describe for the first time two cases of a peculiar form of oropharyngeal dystonia induced not by a specific motor skill but by swallowing specific foods only when presented with specific consistencies or texture.

## Case Report

### Case 1

**Figure 1:**
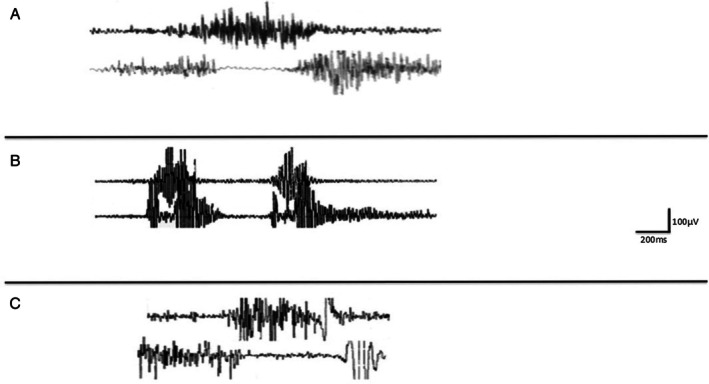
Patient 1: IC (top) and cricopharyngeal (CP) (bottom) electromyography‐recording. (A) normal CP/IC coordination with CP relaxation while swallowing chopped tomato. (B) CP/IC dystonic incoordination during swallowing tomato sauce. There is a IC muscle split associated with uncoordinated contraction, indicating a premature interruption of physiological peristalsis followed and preceded by a spasm‐like activity. The CP pause appears mainly delayed but also slightly reduced in duration. (C) Forty‐eight hours after treatment: restoration of normal coordination. Sweep: 200 ms/Div; amplitude: 100 μV/Div.

A 28‐year‐old woman with negative anamnestic or family history for neurological/psychiatric disorders was referred to our clinic because of the onset of dysphagia over the previous 2 months, which occurred only while swallowing tomato sauce alone or mixed with any food, but not chopped tomatoes. She never complained of difficulty swallowing other foods of any consistency or texture. Physical, neurological, cranial nerve examination, allergology test for tomato intake as well as rhino‐fiberscope, esophago‐gastroscopic, pH‐metric examinations, chest/abdominal CT, brain, cervical/neck MRI, nerve conduction studies, routine and cranial districts electromyography (EMG) were normal. Laryngeal sensibility, tested by endoscopic evaluation of the laryngeal adductor reflex (LAR) by using “air puff” was normal. When we evaluated the coordination between voluntary and involuntary pharyngeal muscles by simultaneous EMG of the cricopharyngeal (CP) muscle of the upper esophageal sphincter (UES), and inferior pharyngeal constrictor (IC) muscle while swallowing different bolus sizes and food consistencies including chopped tomato, a normal IC/CP coordination with UES hyperactivity was observed (Fig. 1A).^5^, ^6^, ^7^ Conversely, when this examination was performed during swallowing tomato sauce, an IC/CP dyscoordination with delayed and reduced cricopharyngeal relaxation was found (Fig. 1B).

Video‐fluoroscopy (VFS) with three different food consistencies (thin liquid, semi‐solid or solid) of standardized bolus size, including chopped tomato was performed, revealing normal swallowing. Conversely, when tomato sauce mixed with barium was tested, VFS showed a reduced pharyngeal clearance and an incomplete UES opening without oral‐phase abnormalities. Dysphagia severity was scored using the Penetration/Aspiration Scale (PAS).^8^ A severe dysphagia (score: 5) with post‐deglutitive penetration was observed after swallowing tomato sauce. Conversely no dysphagia (score: 1) was present after swallowing the other different consistencies, including chopped tomato.

### Case 2

A 23‐year‐old woman with a negative history for neurological/psychiatric disorders, experienced in the previous 6 months, similar symptoms to the patient reported above, but only while she tried to swallow chopped pineapple alone and/or mixed with other foods, while she had no difficulty with other foods including pineapple juice. Allergology tests, physical examination rhino‐fibroscopy, esophago‐gastroscopy, pH‐metry, chest/abdominal CT scan, laryngeal sensory tests, neurophysiological examinations, brain and cervical/neck MRI were normal.

VFS and pharyngeal EMG performed while swallowing different bolus sizes and consistencies including pineapple juice was normal.^5^, ^6^, ^7^ Conversely, both these examinations showed an IC/CP dyscoordination with UES hyperactivity while swallowing chopped pineapple with post‐deglutitive penetration (PAS score: 4).

**Figure 2:**
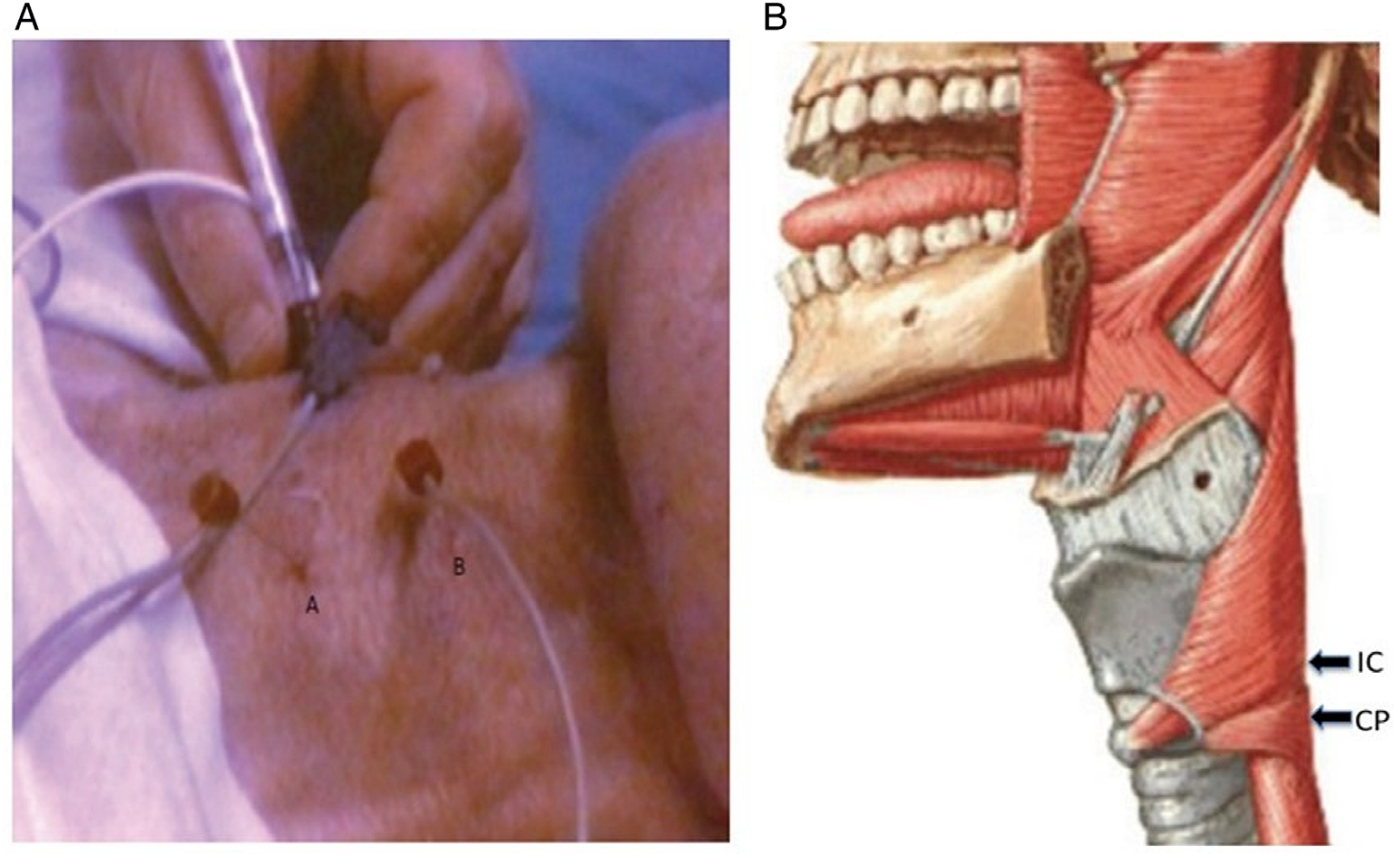
Botulinum toxin (BTX) injection (patients 1). Left: BTX was injected into the right cricopharyngeal muscle. The EMG activity of the CP muscle (A) and the inferior pharyngeal constrictor muscle (B) were simultaneously recorded. Right: anatomical representation of the CP and IC muscles.

In both patients, a peculiar form of pharyngeal FTSD was hypothesized and botulinum toxin (BTX) treatment for UES dysfunction was proposed.^5^, ^6^, ^7^ *Incobotulinum Toxin A* (10 U; dilution 200 μL) was injected into the cricopharyngeal muscle (one site for each patient) under electromyographic control using the previously reported technique^5^, ^6^, ^7^ (Fig. 2). The treatment was administered off‐label, prior acquisition of informed consent.

After 7 days, both patients were able to safely swallow tomato sauce and chopped pineapple, respectively. EMG and VFS showed the recurrence of physiological swallowing. The effect lasted approximately 4 months. Thereafter, symptoms reoccurred, and both patients requested further injections, which restored normal function.

## Discussion

In these patients, a peculiar form of pharyngeal dystonia, selectively affecting the ability to swallow a specific food only when it is presented with a specific consistency or texture may be hypothesized. To our knowledge this is the first report of pharyngeal dystonia implying sensitivity not so much for specific foods but rather for their specific consistency/texture. The mechanism is unknown. Basal ganglia are both directly and indirectly involved in the strict control of swallowing. The central pattern generators (CPGs) for swallowing coordinate the swallowing process until leading the final stage of swallowing. CPG corresponds to the area of the nucleus tractus solitarius (NTS). The NTS receives fibers from the nucleus ambiguous (NA), which in turn sends efferent fibers to the nuclei of the main cranial nerve involved in swallowing.^9^ Also, NTS receives sensitive afferences from oral, pharyngeal, and laryngeal mucosa as well as from upper cerebral areas, including the basal ganglia and can modulate swallowing dependent on bolus properties such as size, consistency, texture, and temperature.^9^ Since different food consistencies and textures require engagement of different cortical and subcortical structures,^10^ a role of a selective dysfunction of the sensorimotor pathway involving the basal ganglia‐thalamic circuit and leading to defective pharyngeal swallowing control may be hypothesized. This pathophysiological mechanism of impaired sensorimotor integration is like that reported for FTSDs, but differently from FTSD, UES dysfunction may be exclusively triggered by food consistency/texture rather than by a specific task.

## Author Roles

(1) Clinical Evaluation: A. Neurological examination B. ENT examination; (2) Instrumental Evaluation: A. Pharyngeal EMG evaluation B. Videofluoroscopy C. Rhino‐fibyoscopy; (3) Manuscript Preparation: A. Writing of the first draft, B. Review and Critique.

D.A.R.: 1A, 2B, 3A.

D.B.: 3B, 2C.

A.A.: 1A, 3B.

E.A.: 2B, 3A.

R.M.‐R.: 1B, 2B, 2C, 3B.

A.Q.: 1A, 3B.

## Disclosures

**Ethical Compliance Statement:** All submissions, regardless of type, require an Ethical Compliance Statement at the end of the manuscript. This must include all three of the following: The ARNAS “Garibaldi” Ethical Committee approved the study. A written informed consent was obtained by the patients who were clearly and diffusely elucidated about risks/benefits of the clinical and instrumental examinations and treatment. We confirm that all the authors have read the Journal's position on issues involved in ethical publication and affirm that this work is consistent with those guidelines.

**Funding Sources and Conflict of Interest:** No specific funding was received for this work and the authors declare that there are no conflicts of interest relevant to this work.

**Financial Disclosures for the previous 12 months:** The authors declare that there are no additional disclosures to report.

## Data Availability

The data that support the findings of this study are available on request from the corresponding author. The data are not publicly available due to privacy or ethical restrictions.
